# Supplementary material for: A scalable, clinically severe pig model for Duchenne muscular dystrophy
Source: Dis Model Mech. 2021 Dec 16;14(12):dmm049285. doi: 10.1242/dmm.049285 (PMC8688409; doi:10.1242/dmm.049285)
Supplement: Supplementary information [file dmm-14-049285-s1.pdf]

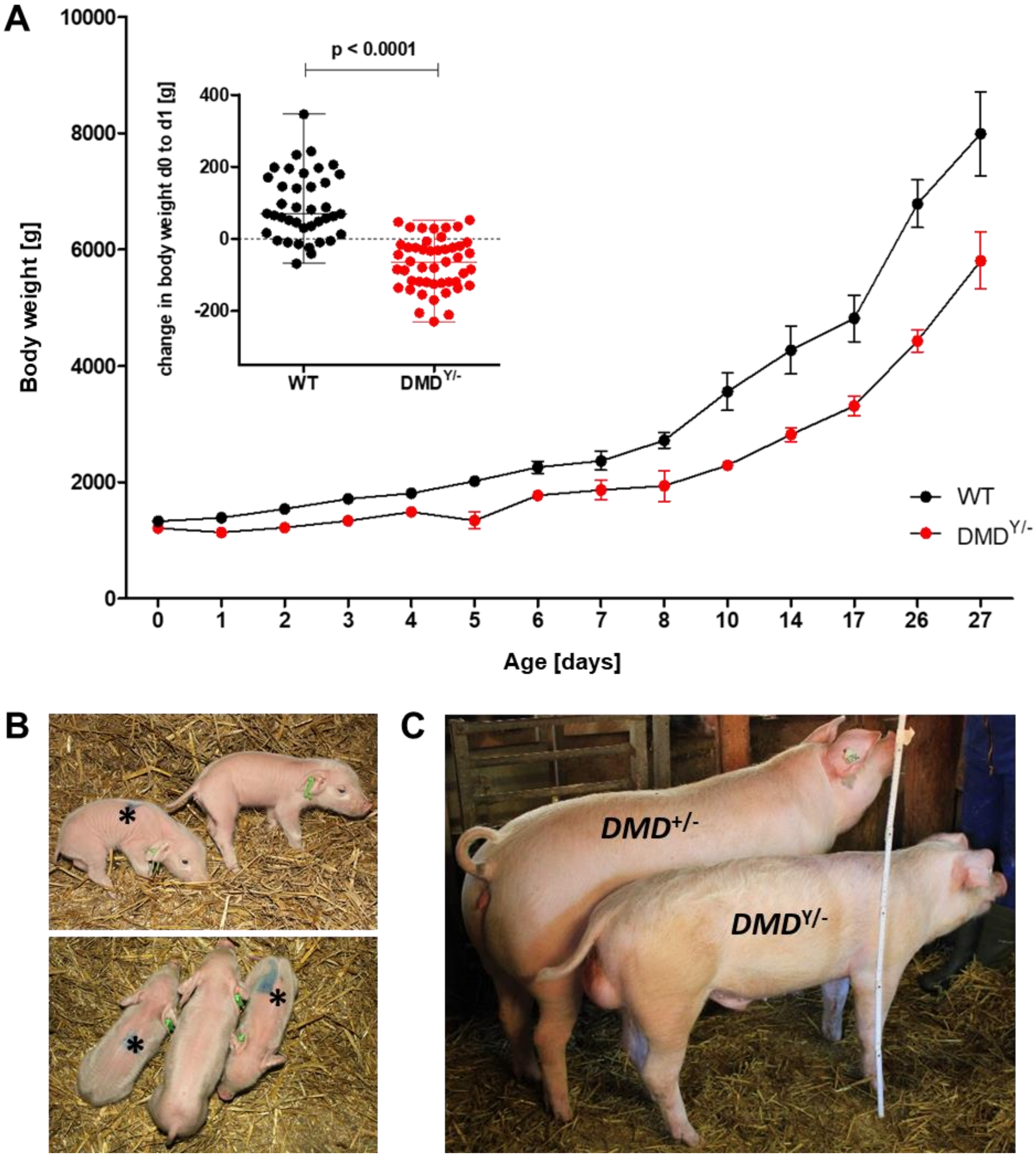

Fig. S1. Reduced birth weight and weight gain of *DMD*$^{Y/-}$ piglets. **A**) Body weights of *DMD*$^{Y/-}$ pigs and male wild-type (WT) littermates in the first 27 days of life (n ≥ 16 per genotype until day 5; subsequently n ≥ 5 per genotype). For the period after day 8, data were adjusted by linear interpolation to the respective ages shown in the graph. Insert: Change in body weight of *DMD*$^{Y/-}$ piglets (n = 47) and male WT littermates (n = 39) between birth and age 24 hours. **B**) Representative pictures of 4-day-old *DMD*$^{Y/-}$ piglets (marked with asterisks) and a male WT littermate. **C**) Adolescent *DMD*$^{Y/-}$ pig #6790 and *DMD*$^{+/-}$ littermate at age 226 days.

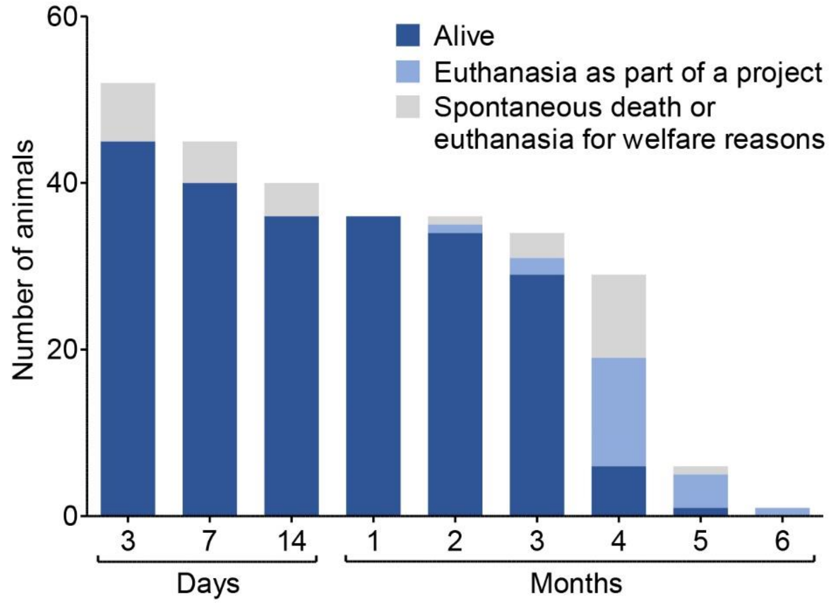

Fig. S2. Reduced neonatal lethality of *DMD*$^{Y/-}$ piglets after optimisation of litter management (cohort of pigs born after 01/2020).

Disease Models & Mechanisms • Supplementary information

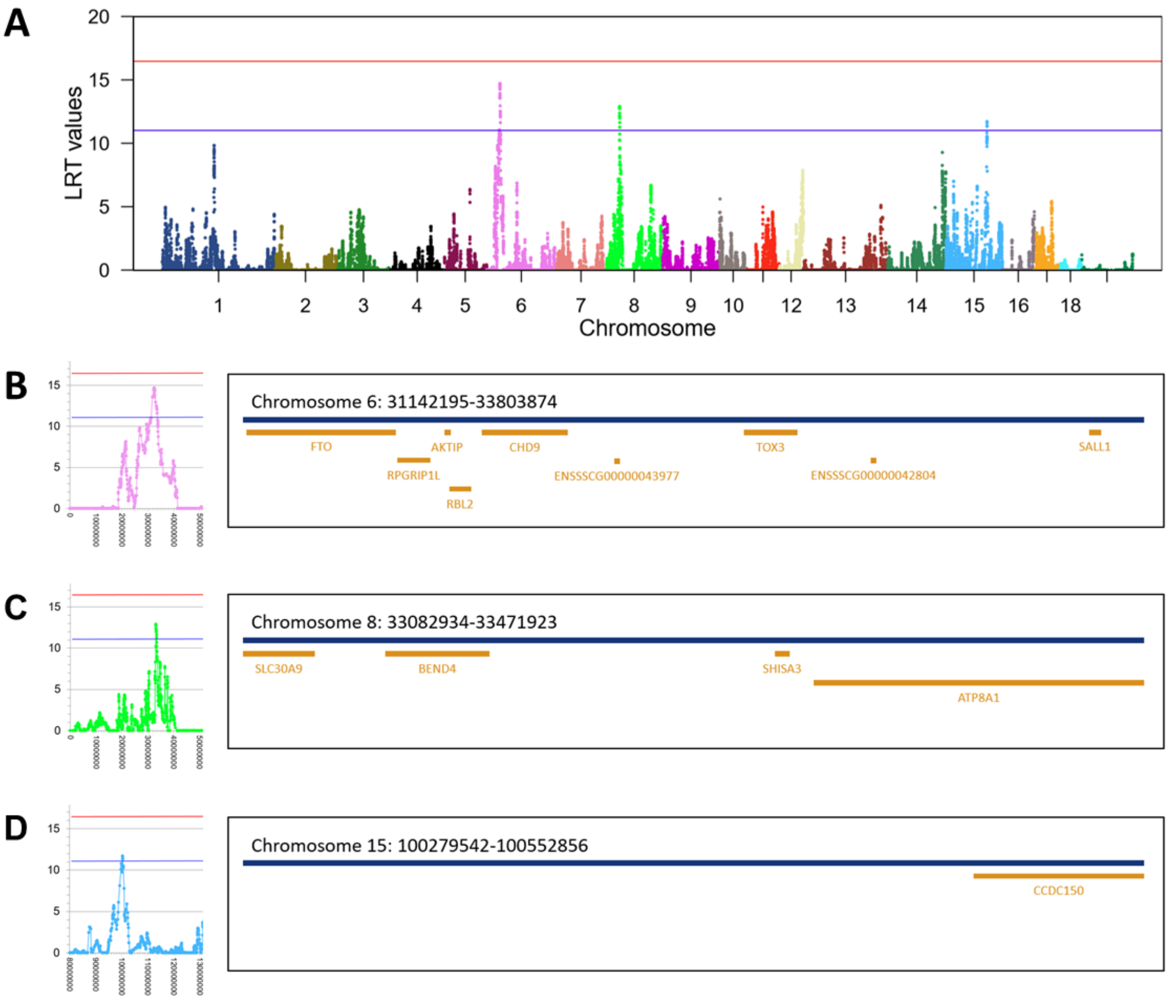

E

Gene	Description	heart	skeletal muscle
FTO	Alpha-ketoglutarate-dependent dioxygenase	+++	++
RPGRIP1L	RPGRIP1 like	++	+
AKTIP	AKT interacting protein	+++	+++
RBL2	RB transcriptional corepressor like 2	++++	++++
CHD9	Chromodomain helicase DNA binding protein 9	+++++	+++++
ENSSSCG00000043977		-	-
TOX3	TOX high mobility group box family member 3	-	-
ENSSSCG00000042804		-	-
SALL1	Spalt like transcription factor 1	+	-
SLC30A9	Solute carrier family 30 member 9	+++	++
BEND4	BEN domain containing 4	-	-
SHISA3	Shisa family member 3	+	+
ATP8A1	ATPase phospholipid transporting 8A1	++	++++
CCDC150	Coiled-coil domain containing 150	++	++

Disease Models & Mechanisms • Supplementary information

Fig. S3. Mapping of quantitative trait loci affecting the life expectancy of *DMD*$^{Y/-}$ pigs. **A**) The association between haplotypes and phenotypes (life expectancy) is tested using the likelihood ratio test statistic (*LRT*). The *LRT* values are shown on the y-axis and the tested positions on the pig chromosomes on the x-axis. Blue horizontal line indicates the level of chromosome-wide significance, red line the level of genome-wide significance. **B**-**C**) QTL regions and protein-coding genes in these regions on chromosomes 6 (**B**), 8 (**C**), and 15 (**D**). **E**) Genes in the candidate regions and their expression in heart and skeletal muscle. Expression data are from the "Pig RNA Atlas" (www.rnaatlas.org) and the "Expression Atlas" (www.ebi.ac.uk). In the PIG RNA Atlas, transcripts per million (TPM) values are calculated per each sample for all protein coding genes, referred to as pTPM. Samples of the same tissue type are then aggregated by using the average pTPM per gene, and resulting values are sample-wise corrected using trimmed mean of M values (TMM) and then gene-wise pareto scaled, resulting in an expression score referred to as NX. Asterisks indicate ranges of NX values: * <5; ** 5–10; *** >10–15; **** >15–20; ***** >20–25.

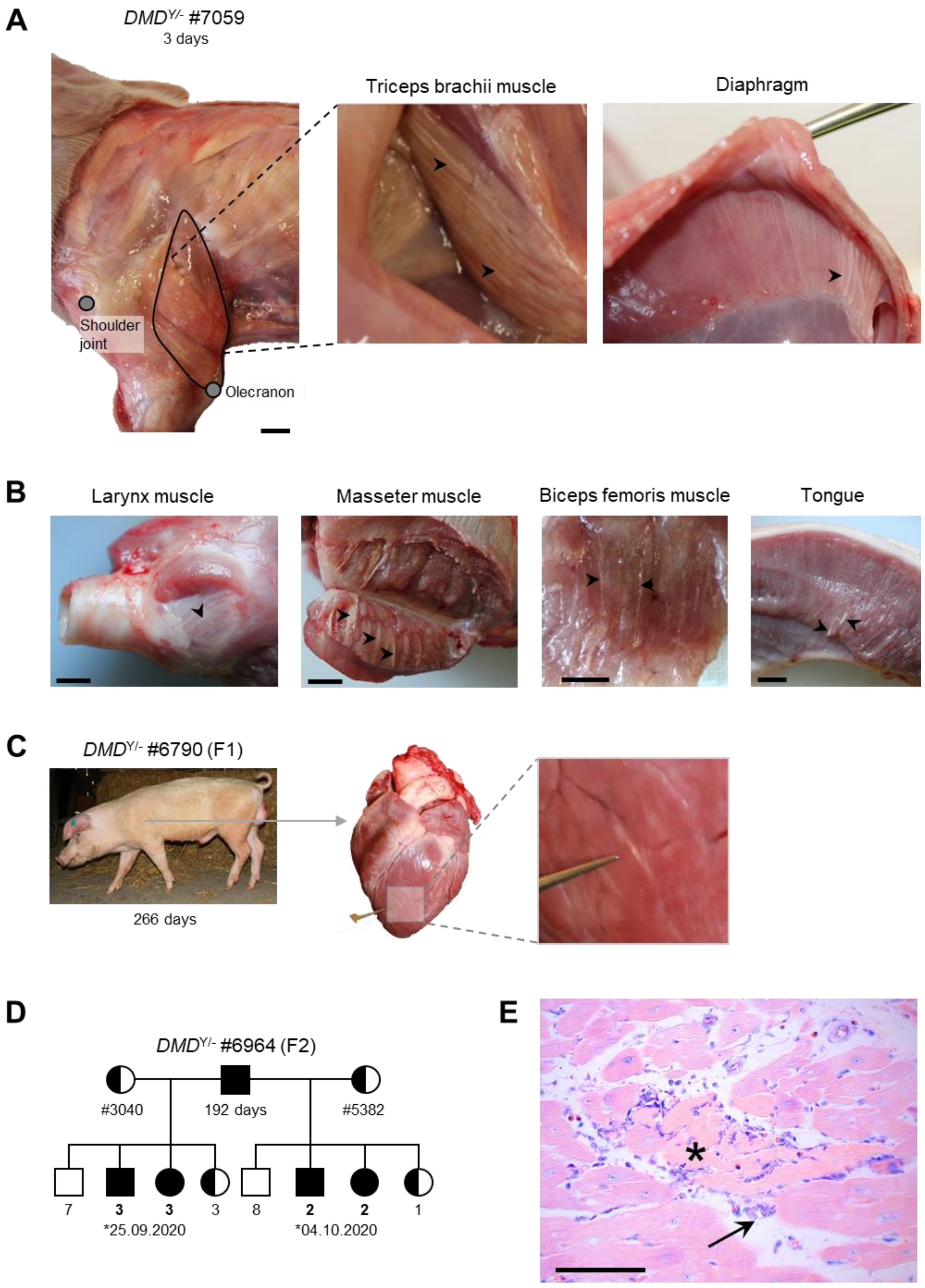

Disease Models & Mechanisms • Supplementary information

Fig. S4. Pathological changes of skeletal muscle and heart in *DMD*$^{Y/-}$ pigs surviving less than one week (**A**), 3-4 months (**B**) or more than 6 months (**C**). **A**) Short-term survivors showed prominent macroscopic alterations of various skeletal muscles, such as streaky white muscle fiber degeneration. Scale bar = 1 cm. **B**) In *DMD*$^{Y/-}$ pigs surviving for 3-4 months, multiple skeletal muscles showed macroscopic signs of degeneration, whereas the heart was macroscopically inconspicuous. Scale bar = 1 cm. **C**) The longest surviving *DMD*$^{Y/-}$ boar #6790 displayed macroscopic myocardial lesions. **D**) From another long-term surviving *DMD*$^{Y/-}$ boar (#6964), semen could be recovered and used for insemination of *DMD*$^{+/-}$ carrier sows, which gave birth to offspring, including *DMD*$^{+/-}$ and *DMD*$^{-/-}$. **E**) Histopathological lesions in myocardium (left ventricle) of the long-surviving (192 days) *DMD*$^{Y/-}$ boar #6964, displaying inflammatory cell infiltrations (arrows) and necrotic cardiomyocytes (asterisks). Histology: paraffin sections, Giemsa staining. Scale bar: 50 µm.

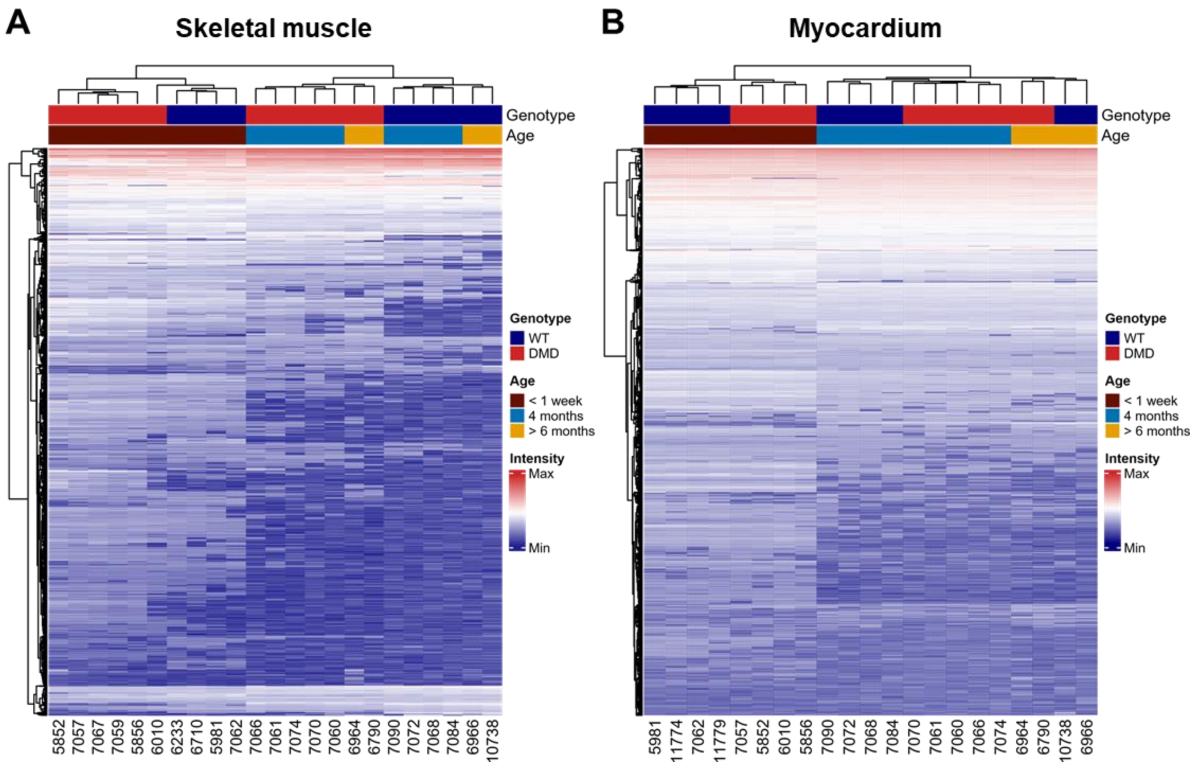

Fig. S5. Unsupervised hierarchical clustering of normalized protein intensities from skeletal muscle (**A**) and myocardium (**B**) of < 1 week old, 4 months old, and > 6 months old WT and *DMD*$^{Y/-}$ animals**.**

Disease Models & Mechanisms • Supplementary information

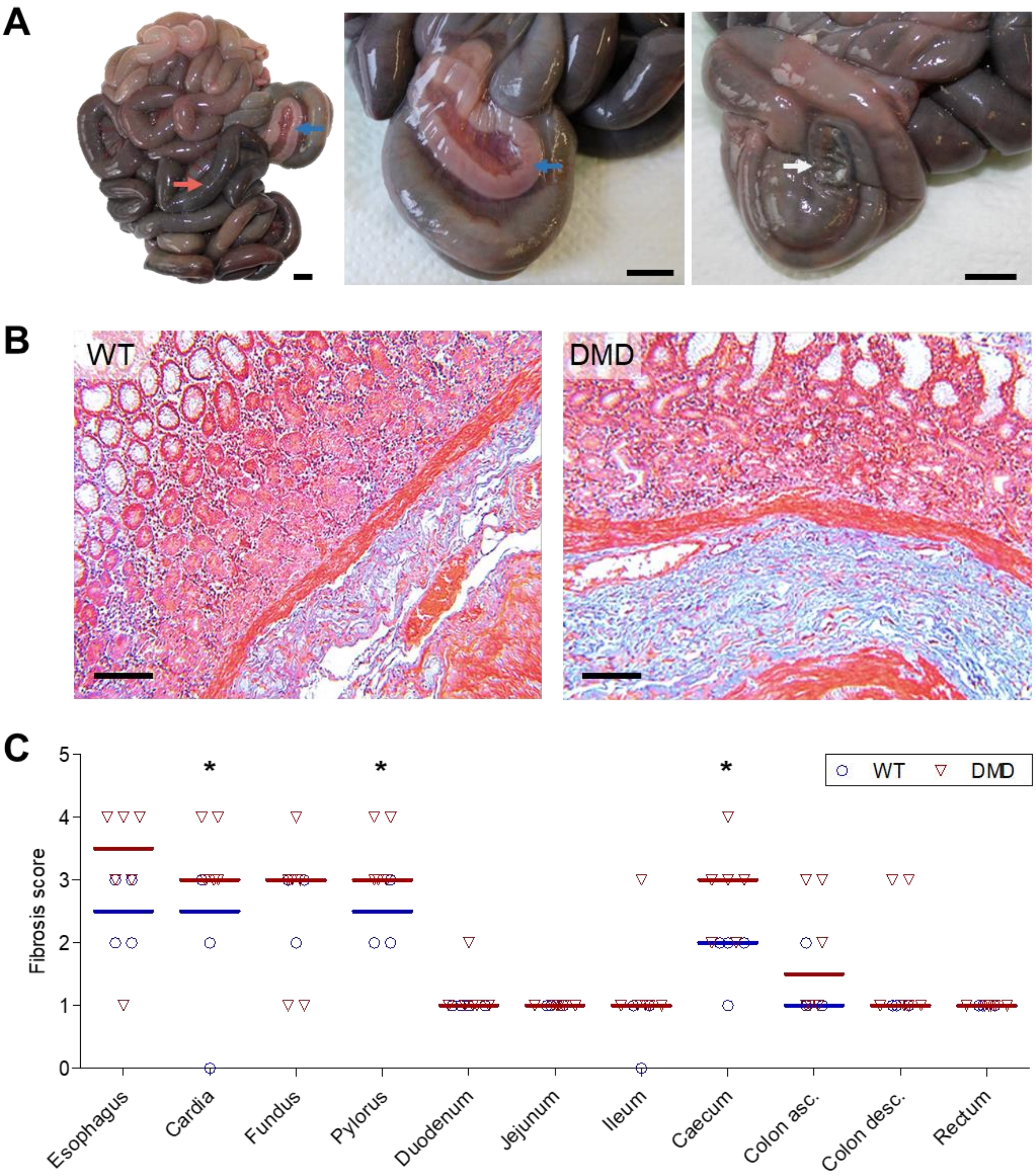

Fig. S6. Macroscopic and histological alterations of the gastrointestinal tract in *DMD*$^{Y/-}$ pigs. **A**) Macroscopic findings in a 6-day-old *DMD*$^{Y/-}$ piglet showing clinical symptoms of a gastrointestinal phenotype. Obstruction of the large intestine (red arrow), empty intestine section subsequent to the obstructed part (blue arrow), peak of the ascending large intestine with porous site (white arrow). Bars = 1 cm. **B**) Histology of the stomach cardia from a 4-month-old wild-type (WT) and an age-matched *DMD*$^{Y/-}$ pig. The *DMD*$^{Y/-}$ pig shows an increased deposition of collagenous fibers (green-blue colored). Masson's Trichrome stain. Bar = 250 µm. **C**) Fibrosis scores in different parts of the gastrointestinal tract. 1 = absent; 2 = sub-significant; 3 = mild; 4 = moderate; 5 = severe. Asterisks indicate significant differences between *DMD*$^{Y/-}$ and WT pigs (Mann-Whitney U test, one-sided: $p < 0.05$).

Disease Models & Mechanisms • Supplementary information

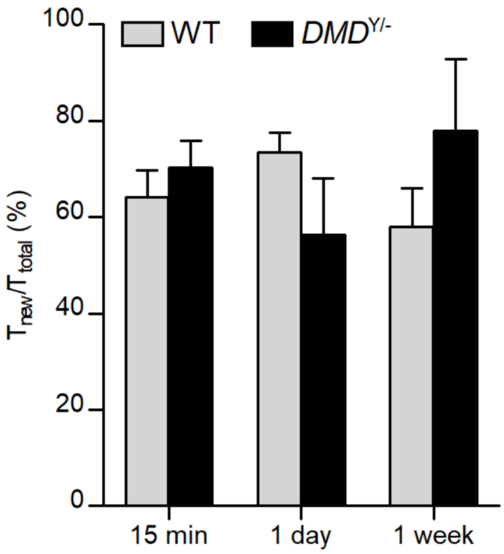

Fig. S7. Proportionate time (T_{new}/T_{total}) spent by the pigs investigating the new object (T_{new}) relative to the total exploration time for both objects (T_{total}), during the 5 min in the trial box. Both groups spent more time with the new object and no significant differences between the groups were found.

Disease Models & Mechanisms • Supplementary information

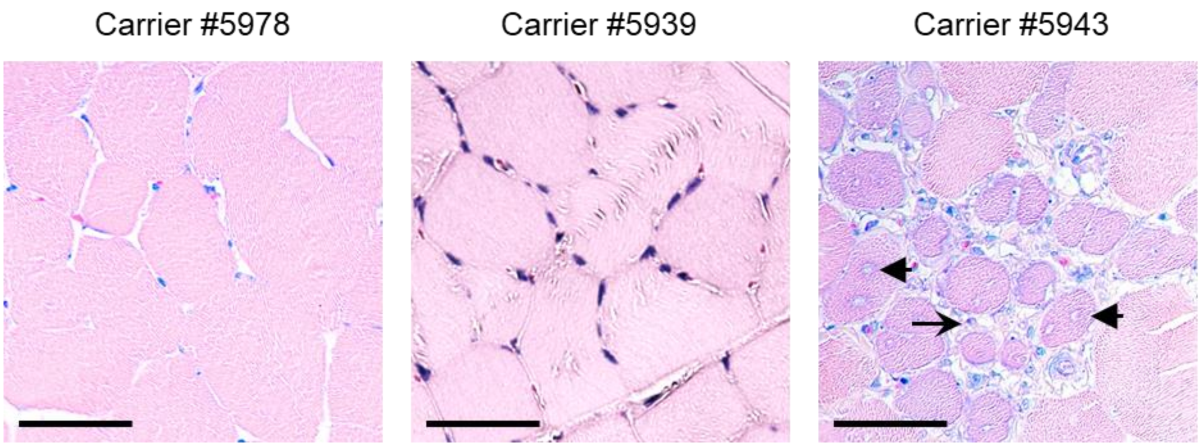

Fig. S8. Variable histological alterations of skeletal muscle (triceps brachii) in 6-month-old *DMD*$^{+/-}$ carrier pigs. Histopathological alterations are indicated (arrow: inflammatory cell infiltration comprising macrophages/histiocytes, plasma cells and few lymphocytes; arrowheads: skeletal myocytes with centralized nuclei). Paraffin sections, Giemsa-staining. Bars = 50 µm.

Table S1. Breeding of F1 and F2 *DMD*$^{+/-}$ offspring of founder sow #3040

Gen.	Sow	M/F sow	Boar (WT)	Litter	Birth date	WT m	WT f	*DMD*$^{+/-}$	*DMD*$^{Y/-}$
F1	**#5153**	#3040/Eposo	Isomer	1	2017-10-18	1	6	4	5
			Casanova	2	2018-03-16	1	2	4	5
			Boletto	3	2018-08-09	7	4	2	3
F1	**#5381**	#3040/Rabatz	Costa	1	2018-01-16	4	1	5	2
			Costa	2	2018-06-14	5	0	0	0
			Costa	3	2018-11-08	3	3	6	2
F1	**#5382**	#3040/Rabatz	Isomer	1	2018-03-09	4	4	3	9
			Boletto	2	2018-08-03	1	6	7	4
			Costa	3	2019-03-27	4	8	1	3
			Costa	4	2019-08-22	5	1	5	5
F1	**#5383**	#3040/Rabatz	Costa	1	2017-11-18	1	4	3	6
			Costa	2	2018-04-12	4	2	4	2
			Casanova	3	2018-09-06	3	5	5	3
			Cor	4	2019-10-08	3	2	7	2
F1	**#6314**	#3040/Costa	Rabart	1	2019-12-02	4	3	3	3
			Rabart	2	2020-04-21	0	6	4	2
			Last	3	2021-02-19	4	5	3	2
F1	**#6794**	#3040/Cor	Rabart	1	2020-08-24	5	1	3	5
			Last	2	2021-02-19	2	5	5	1
F2	**#6225**	#5382/Boletto	Cor	1	2019-09-02	0	1	2	1
			Last	2	2020-07-02	4	2	5	5
			Unox	3	2020-11-27	3	3	5	3
F2	**#6243**	#5153/Boletto	Cor	1	2019-06-13	5	3	3	2
			Rabart	2	2020-04-16	3	0	5	7
			Last	3	2020-09-18	5	4	3	2
			Last	4	2021-02-19	5	3	4	3
F2	**#6245**	#5153/Boletto	Rabart	1	2019-06-19	3	4	3	3
			Rabart	2	2019-11-09	2	3	4	7
			Rabal	3	2020-04-02	1	0	3	6
			Rabart	4	2020-09-05	4	2	3	3
			Last	5	2021-02-19	2	5	4	8
**Total**				**31**		**98**	**98**	**118**	**114**

M/F sow = mother/father of the sow; WT m = wild-type male; WT f = wild-type female.

Table S2. Overview of samples collected in the DMD biobank

Organ system	Organ/tissue	Location/tissue compartment	Samples
Central nervous system	Brain	Hippocampus	FFPE, -80°C
		Temporo-ventral body of hippocampus	FFPE, -80°C
		Amygdala	FFPE, -80°C
		Cerebellar cortex	FFPE, -80°C
Cardiovascular system	Heart transmural (epicardium, myocardium, endocardium)	Left ventricle basis	Cryo, FFPE, -80°C
		Left ventricle middle	Cryo, FFPE, -80°C
		Left ventricle apex	Cryo, FFPE, -80°C
		Septum basis	Cryo, FFPE, -80°C
		Septum middle	Cryo, FFPE, -80°C
		Septum apex	Cryo, FFPE, -80°C
		Right ventricle basis	Cryo, FFPE, -80°C
		Right ventricle middle	Cryo, FFPE, -80°C
		Right ventricle apex	Cryo, FFPE, -80°C
		Right atrial appendage	FFPE, -80°C
		Left atrial appendage	FFPE, -80°C
Musculo-skeletal system	Triceps brachii	Central muscle head	Cryo, FFPE, -80°C
	Gluteobiceps	Central muscle head	Cryo, FFPE, -80°C
	Diaphragm	From the left thorax wall	Cryo, FFPE, -80°C
Gastro-intestinal tract	Tongue	Basis/middle/apex	FFPE
	Esophagus		Cryo, FFPE, -80°C
	Stomach	Cardiac region	Cryo, FFPE, -80°C
		Fundus region	Cryo, FFPE, -80°C
		Pylorus region	Cryo, FFPE, -80°C
	Duodenum		Cryo, FFPE, -80°C
	Jejunum		Cryo, FFPE, -80°C
	Ileum		Cryo, FFPE, -80°C
	Caecum		Cryo, FFPE, -80°C
	Ascending colon		Cryo, FFPE, -80°C
	Descending colon		Cryo, FFPE, -80°C
	Rectum		Cryo, FFPE, -80°C
Hepato-pancreatic system	Liver		Cryo, FFPE, -80°C
Uro-genital system	Testis	Left testis	Cryo, FFPE
	Epididymis	Head/Body/Tail	FFPE
	Kidney	Cortex	Cryo, FFPE, -80°C
		Medulla	Cryo, FFPE, -80°C
Respiratory tract	Larynx		FFPE
	Lung		Cryo, FFPE, -80°C
Hematopoietic system	Spleen		Cryo, FFPE, -80°C
Body fluids	Blood serum		-80°C
	Li-heparin blood plasma		-80°C
	EDTA blood plasma		-80°C

Table S3. Proteins Identified and quantified by nano-LC-MS/MS-based proteomics in myocardium

Click here to download Table S3

Disease Models & Mechanisms • Supplementary information

Table S4. Overview of samples collected in the carrier biobank

Organ system	Organ/tissue	Location/tissue compartment	Samples
Cardiovascular system	Heart transmural (epicardium, myocardium, endocardium)	Left ventricle	Cryo, FFPE, -80°C
		Septum	Cryo, FFPE, -80°C
		Right ventricle	Cryo, FFPE, -80°C
Musculo-skeletal system	Triceps brachii	Central muscle head	Cryo, FFPE, -80°C
	Gluteobiceps	Central muscle head	Cryo, FFPE, -80°C
	Diaphragm	From the left thorax wall	Cryo, FFPE, -80°C
	Longissimus dorsi	Central muscle head	Cryo, FFPE, -80°C
Body fluids	Blood serum		-80°C
	Li-heparin blood plasma		-80°C
	EDTA blood plasma		-80°C
	Urine		-80°C

Disease Models & Mechanisms • Supplementary information

Table S5. Haplo-/diplotypes at the QTL on chr. 6 affecting life expectancy of DMD pigs

PhC = Phenotype (life expectancy in days); DipC = Diplotype; DipE = estimated effect of diplotype

Haplotypes/diplotypes with positive and negative effects are shaded in green and red, respectively.

Nr	TierLID	mHap|____.____|____X____|____.____|____0	pHap|____.____|____X____|____.____|____0	HapC (m)	HapC (p)	PhC	DipC	DipE	PolygenE
44	DMD6828	AGAAGAACCGGAAAGAAGAGGCGGGGGAAGAAAGGAGGAC	GAACACGAAGGAAAGGGAAGAAGGGGAAAAGGAGGGAGGC	3	17	9	26	-40	0,000010
32	DMD6293	AGAAGAACCGGAAAGAAGAGGCGGGGGAAGAAAGGAGGAC	GACAGCGACGGAGAGGAGGAGAGGGGAAAGAAAGGGAAGC	3	14	2	20	-36	-0,000010
30	DMD6235	AGAAAAGACGGAGAAGGAGAGAGGGGAAAGAAGAGGGAAC	GAAAGAAAAGGAGGGAAAGGGAAAAAAGGAGGGAGAAAAA	13	5	6	19	-33	-0,000030
31	DMD6238	GAAAGAAAAGGAGGGAAAGGGAAAAAAGGAGGGAGAAAAA	AGAAAAGACGGAGAAGGAGAGAGGGGAAAGAAGAGGGAAC	5	13	3	19	-33	-0,000030
53	DMD7057	GAAAGAAAAGGAGGGAAAGGACAGGAGAAAGGAGGGAAGC	GAAAGAAAAGGAGGGAAAGGGAAAAAAGGAGGGAGAAAAA	8	5	5	30	-15	-0,000030
55	DMD7059	GAAAGAAAAGGAGGGAAAGGGAAAAAAGGAGGGAGAAAAA	AGAAGAAAAGGAGGGAAAGGACAGGAGAAAGGAGGGAAGC	5	19	3	32	-15	-0,000020
46	DMD6831	GACAGAACAGAAGGGGAAGGACAAAAGAAGAAAGGGAGGC	GAACACGAAGGAAAGGGAAGAAGGGGAAAAGGAGGGAGGC	6	17	25	27	-7,6	-0,000010
47	DMD6836	GAACACGAAGGAAAGGGAAGAAGGGGAAAAGGAGGGAGGC	GACAGAACAGAAGGGGAAGGACAAAAGAAGAAAGGGAGGC	17	6	6	27	-7,6	-0,000020
48	DMD6837	GACAGAACAGAAGGGGAAGGACAAAAGAAGAAAGGGAGGC	GAACACGAAGGAAAGGGAAGAAGGGGAAAAGGAGGGAGGC	6	17	4	27	-7,6	0,000000
42	DMD6755	GACAGAACAGAAGGGGAAGGACAAAAGAAGAAAGGGAGGC	GAAAGAAAAGGAGGGAAAGGGAAAAAAGGAGGGAGAAAAA	6	5	24	24	-4,6	-0,000010
75	DMD7258	GACAGAACAGAAGGGGAAGGACAAAAGAAGAAAGGGAGGC	GAAAGAAAAGGAGGGAAAGGGAAAAAAGGAGGGAGAAAAA	6	5	94	24	-4,6	0,000010
10	DMD5721	GACAGAACAGAAGGGGAAGGACAAAAGAAGAAAGGGAGGC	AGAAGAACCGGAAAGAAGAGGCGGGGGAAGAAAGGAGGAC	6	3	4	6	-3,3	0,000010
15	DMD5852	AGAAGAACCGGAAAGAAGAGGCGGGGGAAGAAAGGAGGAC	GACAGAACAGAAGGGGAAGGACAAAAGAAGAAAGGGAGGC	3	6	3	6	-3,3	0,000000
16	DMD5856	GACAGAACAGAAGGGGAAGGACAAAAGAAGAAAGGGAGGC	AGAAGAACCGGAAAGAAGAGGCGGGGGAAGAAAGGAGGAC	6	3	3	6	-3,3	0,000000
22	DMD6011	GACAGAACAGAAGGGGAAGGACAAAAGAAGAAAGGGAGGC	AGAAGAACCGGAAAGAAGAGGCGGGGGAAGAAAGGAGGAC	6	3	50	6	-3,3	0,000030
37	DMD6577	AGAAGAACCGGAAAGAAGAGGCGGGGGAAGAAAGGAGGAC	AGAAAAAAAAGGAGGAAAGGACAAAAGAAGAAAGGGAGGC	3	15	71	22	-3,1	0,000000
4	DMD4059	GACAGAACAGAAGGGGAAGGACAAAAGAAGAAGAGGAAAC	AGAAGAACCGGAAAGAAGAGGCGGGGGAAGAAAGGAGGAC	4	3	114	3	-2,8	0,000010
27	DMD6214	AGAAAAGACGGAGAAGGAGAGAGGGGAAAGAAGAGGGAAC	GACAGAACAGAAGGGGAAGGACAAAAGAAGAAAGGGAGGC	13	6	38	17	0,9	-0,000020
28	DMD6215	GACAGAACAGAAGGGGAAGGACAAAAGAAGAAAGGGAGGC	AGAAAAGACGGAGAAGGAGAGAGGGGAAAGAAGAGGGAAC	6	13	3	17	0,9	-0,000040
11	DMD5724	AGAAAAAAAAGGAGGAAAGAGCAAGGAAAGAAAGGGAAAC	AGAAGAACCGGAAAGAAGAGGCGGGGGAAGAAAGGAGGAC	7	3	139	7	2,55	0,000050
21	DMD5984	AGAAAAAAAAGGAGGAAAGAGCAAGGAAAGAAAGGGAAAC	AGAAGAACCGGAAAGAAGAGGCGGGGGAAGAAAGGAGGAC	7	3	102	7	2,55	0,000040
24	DMD6016	AGAAGAACCGGAAAGAAGAGGCGGGGGAAGAAAGGAGGAC	AGAAAAAAAAGGAGGAAAGAGCAAGGAAAGAAAGGGAAAC	3	7	54	7	2,55	0,000020
33	DMD6311	AGAAGAACCGGAAAGAAGAGGCGGGGGAAGAAAGGAGGAC	AGAAAAAAAAGGAGGAAAGAGCAAGGAAAGAAAGGGAAAC	3	7	3	7	2,55	0,000000
35	DMD6411	AGAAAAAAAAGGAGGAAAGAGCAAGGAAAGAAAGGGAAAC	AGAAGAACCGGAAAGAAGAGGCGGGGGAAGAAAGGAGGAC	7	3	121	7	2,55	0,000020
45	DMD6829	AGAAAAAAAAGGAGGAAAGAGCAAGGAAAGAAAGGGAAAC	AGAAGAACCGGAAAGAAGAGGCGGGGGAAGAAAGGAGGAC	7	3	1	7	2,55	-0,000030
8	DMD5139	GAAAGAAAAGGAGGGAAAGGGAAAAAAGGAGGGAGAAAAA	GAACAAGCCAGAAGGGAAGAGAAGGGAAAAGGAGAAAAAA	5	2	69	5	3,89	0,000010
9	DMD5145	GAACAAGCCAGAAGGGAAGAGAAGGGAAAAGGAGAAAAAA	GAAAGAAAAGGAGGGAAAGGGAAAAAAGGAGGGAGAAAAA	2	5	5	5	3,89	-0,000020
19	DMD5970	GACAGCGACGGAGAGGAGGAGAGGGGAAAGAAAGGAGGAC	GAACAAGCCAGAAGGGAAGAGAAGGGAAAAGGAGAAAAAA	12	2	2	13	4,87	-0,000020
80	DMD7310	AGAAGAACCGGAAAGAAGAGGCGGGGGAAGAAAGGAGGAC	GAACAAGCCAGAAGGGAAGAGAAGGGAAAAGGAGAAAAAA	3	2	5	37	5,27	-0,000020
3	DMD4058	GAACACGAAGGGGAAGGAGGACAGGGGAAGAAAGGAAAAC	AGAAGAACCGGAAAGAAGAGGCGGGGGAAGAAAGGAGGAC	1	3	24	2	7,81	-0,000020
5	DMD4070	AGAAGAACCGGAAAGAAGAGGCGGGGGAAGAAAGGAGGAC	GAACACGAAGGGGAAGGAGGACAGGGGAAGAAAGGAAAAC	3	1	23	2	7,81	-0,000020
29	DMD6231	GAAAGAAAAGGAGGGAAAGGGAAAAAAGGAGGGAGAAAAA	AGAAAAGACGGAGAAGGAGGACAGGGGAAGAAAGGAAAAC	5	11	71	18	8,3	-0,000050
39	DMD6707	GAAAGAAAAGGAGGGAAAGGGAAAAAAGGAGGGAGAAAAA	AGAAAAGACGGAGAAGGAGGACAGGGGAAGAAAGGAAAAC	5	11	5	18	8,3	-0,000030
40	DMD6711	GAAAGAAAAGGAGGGAAAGGGAAAAAAGGAGGGAGAAAAA	AGAAAAGACGGAGAAGGAGGACAGGGGAAGAAAGGAAAAC	5	11	26	18	8,3	-0,000030
60	DMD7069	AGAAAAGACGGAGAAGGAGGACAGGGGAAGAAAGGAAAAC	GAAAGAAAAGGAGGGAAAGGGAAAAAAGGAGGGAGAAAAA	11	5	10	18	8,3	-0,000060
62	DMD7074	GAAAGAAAAGGAGGGAAAGGGAAAAAAGGAGGGAGAAAAA	AGAAAAGACGGAGAAGGAGGACAGGGGAAGAAAGGAAAAC	5	11	116	18	8,3	-0,000020
77	DMD7282	GAAAGAAAAGGAGGGAAAGGGAAAAAAGGAGGGAGAAAAA	AGAAAAGACGGAGAAGGAGGACAGGGGAAGAAAGGAAAAC	5	11	39	18	8,3	0,000000
89	DMD7467	AGAAAAGACGGAGAAGGAGGACAGGGGAAGAAAGGAAAAC	GAAAGAAAAGGAGGGAAAGGGAAAAAAGGAGGGAGAAAAA	11	5	68	18	8,3	0,000020
90	DMD7469	GAAAGAAAAGGAGGGAAAGGGAAAAAAGGAGGGAGAAAAA	AGAAAAGACGGAGAAGGAGGACAGGGGAAGAAAGGAAAAC	5	11	3	18	8,3	0,000020
91	DMD7470	GAAAGAAAAGGAGGGAAAGGGAAAAAAGGAGGGAGAAAAA	AGAAAAGACGGAGAAGGAGGACAGGGGAAGAAAGGAAAAC	5	11	95	18	8,3	0,000030
93	DMD7472	GAAAGAAAAGGAGGGAAAGGGAAAAAAGGAGGGAGAAAAA	AGAAAAGACGGAGAAGGAGGACAGGGGAAGAAAGGAAAAC	5	11	115	18	8,3	0,000040
95	DMD7486	GAAAGAAAAGGAGGGAAAGGGAAAAAAGGAGGGAGAAAAA	AGAAAAGACGGAGAAGGAGGACAGGGGAAGAAAGGAAAAC	5	11	86	18	8,3	0,000000
18	DMD5937	AGAAGAACCGGAAAGAAGAGGCGGGGGAAGAAAGGAGGAC	AGAAAAGACGGAGAAGGAGGACAGGGGAAGAAAGGAAAAC	3	11	77	12	9,63	0,000020
25	DMD6212	AGAAGAACCGGAAAGAAGAGGCGGGGGAAGAAAGGAGGAC	AGAAAAGACGGAGAAGGAGGACAGGGGAAGAAAGGAAAAC	3	11	5	12	9,63	-0,000060
78	DMD7306	AGAAAAGACGGAGAAGGAGGACAGGGGAAGAAAGGAAAAC	AGAAGAACCGGAAAGAAGAGGCGGGGGAAGAAAGGAGGAC	11	3	2	12	9,63	-0,000020
81	DMD7320	AGAAAAGACGGAGAAGGAGGACAGGGGAAGAAAGGAAAAC	AGAAGAACCGGAAAGAAGAGGCGGGGGAAGAAAGGAGGAC	11	3	56	12	9,63	-0,000010
17	DMD5936	GACAGAACAGAAGGGGAGAGGCGGGGGAAGAAAGGAGGAC	AGAAAAGACGGAGAAGGAGGACAGGGGAAGAAAGGAAAAC	10	11	136	11	12,3	0,000030
49	DMD6859	GAACACGAAGGGGAGAAAGGACAGGGGAAGAAAGGAAAAC	AGAAAAGACGGAGAAGGAGAGAGGGGAAAGAAGAGGGAAC	18	13	84	28	12,4	0,000020
65	DMD7169	AGAAAAGACGGAGAAGGAGAGAGGGGAAAGAAGAGGGAAC	AGAAAAGACGGAGAAGGAGGACAGGGGAAGAAAGGAAAAC	13	11	109	35	13,9	0,000030
68	DMD7173	AGAAAAGACGGAGAAGGAGGACAGGGGAAGAAAGGAAAAC	AGAAAAGACGGAGAAGGAGAGAGGGGAAAGAAGAGGGAAC	11	13	118	35	13,9	0,000040
85	DMD7390	AGAAAAGACGGAGAAGGAGAGAGGGGAAAGAAGAGGGAAC	AGAAAAGACGGAGAAGGAGGACAGGGGAAGAAAGGAAAAC	13	11	5	35	13,9	-0,000020
12	DMD5725	GACAGAACAGAAGGGGAAGGACAAAAGAAGAAAGGGAGGC	GAAAGAAAAGGAGGGAAAGGACAGGAGAAAGGAGGGAAGC	6	8	107	8	18	0,000030
20	DMD5983	GAAAGAAAAGGAGGGAAAGGACAGGAGAAAGGAGGGAAGC	AGAAAAAAAAGGAGGAAAGAGCAAGGAAAGAAAGGGAAAC	8	7	123	14	23,7	0,000040
51	DMD6967	AGAAAAAAAAGGAGGAAAGAGCAAGGAAAGAAAGGGAAAC	GAAAGAAAAGGAGGGAAAGGACAGGAGAAAGGAGGGAAGC	7	8	46	14	23,7	-0,000020
34	DMD6409	GACAGAACAGAAGGGGAAGGACAAAAGAAGAAAGGGAGGC	GACAGAACAGAAGGGGAAGGACAAAAGAAGAAAGGGAGGC	6	6	75	21	28,8	0,000010
56	DMD7060	AGAAAAGACGGAGAAGGAGGACAGGGGAAGAAAGGAAAAC	GAAAGAAAAGGAGGGAAAGGACAGGAGAAAGGAGGGAAGC	11	8	124	33	30,9	0,000000
71	DMD7230	AGAAAAGACGGAGAAGGAGGACAGGGGAAGAAAGGAAAAC	GAAAGAAAAGGAGGGAAAGGACAGGAGAAAGGAGGGAAGC	11	8	95	33	30,9	0,000030
73	DMD7232	GAAAGAAAAGGAGGGAAAGGACAGGAGAAAGGAGGGAAGC	AGAAAAGACGGAGAAGGAGGACAGGGGAAGAAAGGAAAAC	8	11	2	33	30,9	-0,000010
38	DMD6649	GACAGAACAGAAGGGGAAGGACAAAAGAAGAAAGGAGGAC	AGAAAAAAAAGGAGGAAAGAGCAAGGAAAGAAAGGGAAAC	16	7	96	23	34,7	0,000020
13	DMD5763	AGAAGAACCGGAAAGAAAGAGAAGGGAAAAGGAGAAAAAA	GACAGAACAGAAGGGGAAGGACAAAAGAAGAAAGGGAGGC	9	6	82	9	36,3	0,000000
14	DMD5769	GAACAAGCCAGAAGGGAAGAGAAGGGAAAAGGAGAAAAAA	GACAGAACAGAAGGGGAAGGACAAAAGAAGAAAGGGAGGC	2	6	108	10	37,4	0,000010
36	DMD6575	GACAGAACAGAAGGGGAAGGACAAAAGAAGAAAGGGAGGC	GAACAAGCCAGAAGGGAAGAGAAGGGAAAAGGAGAAAAAA	6	2	123	10	37,4	0,000020
70	DMD7228	GAACAAGCCAGAAGGGAAGAGAAGGGAAAAGGAGAAAAAA	GACAGAACAGAAGGGGAAGGACAAAAGAAGAAAGGGAGGC	2	6	125	10	37,4	0,000030
72	DMD7231	GAACAAGCCAGAAGGGAAGAGAAGGGAAAAGGAGAAAAAA	GACAGAACAGAAGGGGAAGGACAAAAGAAGAAAGGGAGGC	2	6	74	10	37,4	0,000010
74	DMD7233	GACAGAACAGAAGGGGAAGGACAAAAGAAGAAAGGGAGGC	GAACAAGCCAGAAGGGAAGAGAAGGGAAAAGGAGAAAAAA	6	2	111	10	37,4	0,000030
82	DMD7327	GAACAAGCCAGAAGGGAAGAGAAGGGAAAAGGAGAAAAAA	GACAGAACAGAAGGGGAAGGACAAAAGAAGAAAGGGAGGC	2	6	116	10	37,4	0,000010
7	DMD4796	GACAGAACAGAAGGGGAAGGACAAAAGAAGAAGAGGAAAC	GAACAAGCCAGAAGGGAAGAGAAGGGAAAAGGAGAAAAAA	4	2	97	4	37,7	-0,000010
26	DMD6213	GACAGAACAGAAGGGGAAGGACAAAAGAAGAAAGGGAGGC	AGAAAAGACGGAGAAGGAGGACAGGGGAAGAAAGGAAAAC	6	11	3	16	41,7	-0,000050
41	DMD6744	AGAAAAGACGGAGAAGGAGGACAGGGGAAGAAAGGAAAAC	GACAGAACAGAAGGGGAAGGACAAAAGAAGAAAGGGAGGC	11	6	6	16	41,7	-0,000020
66	DMD7170	AGAAAAGACGGAGAAGGAGGACAGGGGAAGAAAGGAAAAC	GACAGAACAGAAGGGGAAGGACAAAAGAAGAAAGGGAGGC	11	6	98	16	41,7	0,000020
67	DMD7171	GACAGAACAGAAGGGGAAGGACAAAAGAAGAAAGGGAGGC	AGAAAAGACGGAGAAGGAGGACAGGGGAAGAAAGGAAAAC	6	11	98	16	41,7	0,000020
69	DMD7176	AGAAAAGACGGAGAAGGAGGACAGGGGAAGAAAGGAAAAC	GACAGAACAGAAGGGGAAGGACAAAAGAAGAAAGGGAGGC	11	6	105	16	41,7	0,000020
76	DMD7259	AGAAAAGACGGAGAAGGAGGACAGGGGAAGAAAGGAAAAC	GACAGAACAGAAGGGGAAGGACAAAAGAAGAAAGGGAGGC	11	6	192	16	41,7	0,000030
83	DMD7387	AGAAAAGACGGAGAAGGAGGACAGGGGAAGAAAGGAAAAC	GACAGAACAGAAGGGGAAGGACAAAAGAAGAAAGGGAGGC	11	6	118	16	41,7	0,000000
84	DMD7389	GACAGAACAGAAGGGGAAGGACAAAAGAAGAAAGGGAGGC	AGAAAAGACGGAGAAGGAGGACAGGGGAAGAAAGGAAAAC	6	11	118	16	41,7	0,000000
23	DMD6012	GAACAAGCCAGAAGGGAAGAGAAGGGAAAAGGAGAAAAAA	AGAAAAAAAAGGAGGAAAGAGCAAGGAAAGAAAGGGAAAC	2	7	80	15	43,1	0,000000
79	DMD7307	GAACAAGCCAGAAGGGAAGAGAAGGGAAAAGGAGAAAAAA	GAACAAGCCAGAAGGGAAGAGAAGGGAAAAGGAGAAAAAA	2	2	11	36	45,8	-0,000040
64	DMD7082	AGAAAAAAAAGGAGGAAAGAGCAAGGAAAGAAAGAAAAAA	AGAAAAGACGGAGAAGGAGGACAGGGGAAGAAAGGAAAAC	20	11	118	34	47,3	-0,000010
52	DMD6975	AGAAAAAAAAGGAGGAAAGAGCAAGGAAAGAAAGGGAAAC	AGAAAAGACGGAGAAGGAGGACAGGGGAAGAAAGGAAAAC	7	11	5	29	47,4	-0,000030
97	DMD7500	AGAAAAGACGGAGAAGGAGGACAGGGGAAGAAAGGAAAAC	AGAAAAAAAAGGAGGAAAGAGCAAGGAAAGAAAGGGAAAC	11	7	137	29	47,4	0,000050
98	DMD7503	AGAAAAAAAAGGAGGAAAGAGCAAGGAAAGAAAGGGAAAC	AGAAAAGACGGAGAAGGAGGACAGGGGAAGAAAGGAAAAC	7	11	72	29	47,4	0,000020
1	DMD4055	GAACACGAAGGGGAAGGAGGACAGGGGAAGAAAGGAAAAC	GAACAAGCCAGAAGGGAAGAGAAGGGAAAAGGAGAAAAAA	1	2	66	1	48,4	-0,000020
2	DMD4056	GAACACGAAGGGGAAGGAGGACAGGGGAAGAAAGGAAAAC	GAACAAGCCAGAAGGGAAGAGAAGGGAAAAGGAGAAAAAA	1	2	104	1	48,4	-0,000010
6	DMD4794	GAACAAGCCAGAAGGGAAGAGAAGGGAAAAGGAGAAAAAA	GAACACGAAGGGGAAGGAGGACAGGGGAAGAAAGGAAAAC	2	1	97	1	48,4	-0,000010
43	DMD6790	AGAAAAGACGGAGAAGGAGGACAGGGGAAGAAAGGAAAAC	GAACAAGCCAGAAGGGAAGAGAAGGGAAAAGGAGAAAAAA	11	2	266	25	50,4	0,000060
50	DMD6964	GAACAAGCCAGAAGGGAAGAGAAGGGAAAAGGAGAAAAAA	AGAAAAGACGGAGAAGGAGGACAGGGGAAGAAAGGAAAAC	2	11	192	25	50,4	0,000000
63	DMD7081	AGAAAAGACGGAGAAGGAGGACAGGGGAAGAAAGGAAAAC	GAACAAGCCAGAAGGGAAGAGAAGGGAAAAGGAGAAAAAA	11	2	114	25	50,4	-0,000010
86	DMD7439	GAACAAGCCAGAAGGGAAGAGAAGGGAAAAGGAGAAAAAA	AGAAAAGACGGAGAAGGAGGACAGGGGAAGAAAGGAAAAC	2	11	112	25	50,4	0,000040
54	DMD7058	AGAAAAGACGGAGAAGGAGGACAGGGGAAGAAAGGAAAAC	AGAAAAGACGGAGAAGGAGGACAGGGGAAGAAAGGAAAAC	11	11	109	31	54,6	-0,000010
57	DMD7061	AGAAAAGACGGAGAAGGAGGACAGGGGAAGAAAGGAAAAC	AGAAAAGACGGAGAAGGAGGACAGGGGAAGAAAGGAAAAC	11	11	124	31	54,6	-0,000010
58	DMD7066	AGAAAAGACGGAGAAGGAGGACAGGGGAAGAAAGGAAAAC	AGAAAAGACGGAGAAGGAGGACAGGGGAAGAAAGGAAAAC	11	11	116	31	54,6	-0,000030
59	DMD7067	AGAAAAGACGGAGAAGGAGGACAGGGGAAGAAAGGAAAAC	AGAAAAGACGGAGAAGGAGGACAGGGGAAGAAAGGAAAAC	11	11	4	31	54,6	-0,000070
61	DMD7070	AGAAAAGACGGAGAAGGAGGACAGGGGAAGAAAGGAAAAC	AGAAAAGACGGAGAAGGAGGACAGGGGAAGAAAGGAAAAC	11	11	116	31	54,6	-0,000030
87	DMD7465	AGAAAAGACGGAGAAGGAGGACAGGGGAAGAAAGGAAAAC	AGAAAAGACGGAGAAGGAGGACAGGGGAAGAAAGGAAAAC	11	11	90	31	54,6	0,000020
88	DMD7466	AGAAAAGACGGAGAAGGAGGACAGGGGAAGAAAGGAAAAC	AGAAAAGACGGAGAAGGAGGACAGGGGAAGAAAGGAAAAC	11	11	112	31	54,6	0,000020
92	DMD7471	AGAAAAGACGGAGAAGGAGGACAGGGGAAGAAAGGAAAAC	AGAAAAGACGGAGAAGGAGGACAGGGGAAGAAAGGAAAAC	11	11	116	31	54,6	0,000030
94	DMD7474	AGAAAAGACGGAGAAGGAGGACAGGGGAAGAAAGGAAAAC	AGAAAAGACGGAGAAGGAGGACAGGGGAAGAAAGGAAAAC	11	11	134	31	54,6	0,000040
96	DMD7488	AGAAAAGACGGAGAAGGAGGACAGGGGAAGAAAGGAAAAC	AGAAAAGACGGAGAAGGAGGACAGGGGAAGAAAGGAAAAC	11	11	81	31	54,6	-0,000010

Disease Models & Mechanisms • Supplementary information

Table S6. Calculation of group sizes required to prove treatment effect on parameters of cardiac function in the DMD pig model (power 0.8; alpha 0.05)

Parameter	Phenotype		Treatment effect			
	**WT**<br>mean ± SD	***DMD***$^{Y/-}$<br>mean ± SD	25%<br>n/group	50%<br>n/group	75%<br>n/group	100%<br>n/group
**LV fractional shortening (%)**	40.86 ± 4.22	30.00 ± 3.65	35	10	5	4
**LV ejection fraction (M-Mode) (%)**	71.14 ± 3.24	58.00 ± 4.97	27	8	5	3
**LV ejection fraction (B-Mode) (%)**	67.00 ± 7.59	51.25 ± 6.65	53	14	7	5
**LV ejection fraction (Simpson) (%)**	71.14 ± 2.48	57.75 ± 6.65	37	10	6	4

Table S7. Calculation of group sizes required to prove treatment effect on behavioral parameters in the DMD pig model (power 0.8; alpha 0.05)

Parameter	Phenotype		Treatment effect			
	**WT**<br>error rate or mean ± SD	***DMD***$^{Y/-}$<br>error rate or mean ± SD	25%<br>n/group	50%<br>n/group	75%<br>n/group	100%<br>n/group
**Black and White Discrimination Test Round 1**	2 /13	6 / 12	543	138	61	34
**Novel Object Recognition Test T_{total} (s) after 1week**	28.03 ± 23.22	10.80 ± 25.12	496	125	56	32

Disease Models & Mechanisms • Supplementary information
